# Multiple sclerosis cortical and WM lesion segmentation at 3T MRI: a deep learning method based on FLAIR and MP2RAGE

**DOI:** 10.1016/j.nicl.2020.102335

**Published:** 2020-06-30

**Authors:** Francesco La Rosa, Ahmed Abdulkadir, Mário João Fartaria, Reza Rahmanzadeh, Po-Jui Lu, Riccardo Galbusera, Muhamed Barakovic, Jean-Philippe Thiran, Cristina Granziera, Merixtell Bach Cuadra

**Affiliations:** a Signal Processing Laboratory (LTS5), École Polytechnique Fédérale de Lausanne, Switzerland; b Medical Image Analysis Laboratory, Center for Biomedical Imaging (CIBM), University of Lausanne, Switzerland; c Department of Radiology, Lausanne University Hospital and University of Lausanne, Switzerland; d University Hospital of Old Age Psychiatry and Psychotherapy, University of Bern, Bern, Switzerland; e Center for Biomedical Image Computing and Analytics at the Department of Radiology, Perelman School of Medicine, University of Pennsylvania, Philadelphia, PA, United States; f Advanced Clinical Imaging Technology, Siemens Healthcare AG, Lausanne, Switzerland; g Translational Imaging in Neurology Basel, Department of Medicine and Biomedical Engineering, University Hospital Basel and University of Basel, Basel, Switzerland; h Neurologic Clinic and Policlinic, Departments of Medicine, University Hospital Basel and University of Basel, Basel, Switzerland

**Keywords:** MS, Multiple sclerosis, CNS, central nervous system, MRI, magnetic resonance imaging, CLs, cortical lesions, WMLs, white matter lesions, CNN, convolutional neural network, FLAIR, fluid-attenuated inversion recovery, MPRAGE, magnetization-prepared rapid acquisition with gradient echo, MP2RAGE, magnetization-prepared 2 rapid acquisition with gradient echo, DIR, double inversion recovery, MRI, Multiple sclerosis, Cortical lesions, Segmentation, CNN, U-Net, MP2RAGE, FLAIR

## Abstract

The presence of cortical lesions in multiple sclerosis patients has emerged as an important biomarker of the disease. They appear in the earliest stages of the illness and have been shown to correlate with the severity of clinical symptoms. However, cortical lesions are hardly visible in conventional magnetic resonance imaging (MRI) at 3T, and thus their automated detection has been so far little explored. In this study, we propose a fully-convolutional deep learning approach, based on the 3D U-Net, for the automated segmentation of cortical and white matter lesions at 3T. For this purpose, we consider a clinically plausible MRI setting consisting of two MRI contrasts only: one conventional T2-weighted sequence (FLAIR), and one specialized T1-weighted sequence (MP2RAGE). We include 90 patients from two different centers with a total of 728 and 3856 gray and white matter lesions, respectively. We show that two reference methods developed for white matter lesion segmentation are inadequate to detect small cortical lesions, whereas our proposed framework is able to achieve a detection rate of 76% for both cortical and white matter lesions with a false positive rate of 29% in comparison to manual segmentation. Further results suggest that our framework generalizes well for both types of lesion in subjects acquired in two hospitals with different scanners.

## Highlights

• Automated segmentation of cortical and white matter lesions in multiple sclerosis.
• A clinically plausible 3T MRI setting based on FLAIR and MP2RAGE sequences.
• Evaluation is done on a large cohort of 90 patients.
• Results show high cortical and white matter lesion segmentation accuracy.
• Our method generalizes across different hospitals and scanners.

## Graphical abstract

**Figure:**
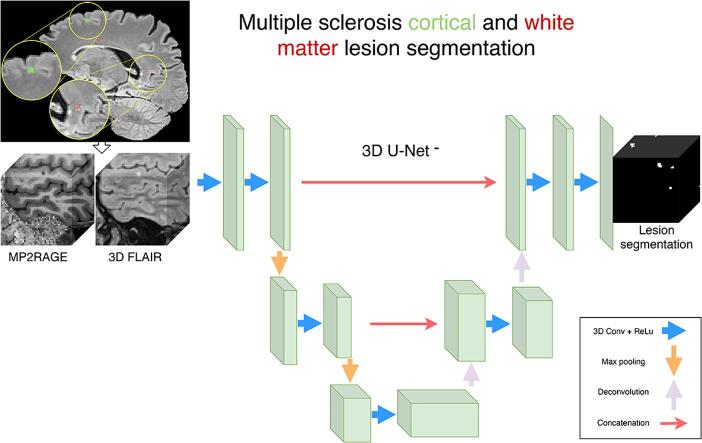

## 1 Introduction

Multiple Sclerosis (MS) is a chronic demyelinating disease involving the central nervous system (CNS). An estimated 2 million people are currently having the disease worldwide (Reich et al., 2018). MS is characterized by sharply delimited lesional areas with primary demyelination, axonal loss, and reactive gliosis, both in the white and in the grey matter. However, the pathological process is not confined to these macroscopically visible focal areas but is generalized in the entire central nervous system (Compston and Coles, 2008, Kuhlmann et al., 2017).

Magnetic resonance imaging (MRI) is the imaging tool of choice to detect such lesions in both the WM and GM of the CNS. The current MS diagnostic criteria (McDonald criteria (Thompson et al., 2018)) are also based on the count and location of lesions in MRI. Common MRI protocols currently include T1-weighted (T1w), T2-weighted (T2w), and fluid-attenuated inversion recovery T2 (FLAIR) sequences. For many years, the main focus in research and clinical practice has been set on white matter lesions (WMLs), clearly visible in the above-mentioned conventional MRI sequences. In the last decade, however, cortical damage has emerged as an important aspect of this disease. Recent studies have shown that the amount and location of cortical lesions (CLs), visible mostly on advanced MRI sequences at high (3T) and ultra-high (7T) magnetic field, correlate better with the severity of the cognitive and physical disabilities than those of WMLs (Calabrese et al., 2009). Since 2017, CLs are also included in the above-mentioned MS diagnostic criteria (Thompson et al., 2018). Consequently, specialized MR sequences with greater sensitivity to detect CLs, such as magnetization-prepared 2 rapid acquisition with gradient echo (MP2RAGE) (Kober et al., 2012, Marques et al., 2010) and double inversion recovery (DIR) (Wattjes et al., 2007), are now more and more used in the clinical setting (Filippi et al., 2019).

Currently, manual segmentation on clinical MRI is considered the gold standard for MS lesion identification and quantification. However, given how time-consuming this process is, several methods for automated MS lesion segmentation have been proposed in the literature (Kaur et al., 2020). These can be broadly classified into supervised and unsupervised approaches. The former ones rely on a manually labeled training set and aim at learning a function that maps the input to the desired output. The latter do not require manual annotations as they are based on generative models that rely on modeling the MRI intensities values of different brain tissues and lesions (Lladó et al., 2012).

Deep learning algorithms are particularly suited for image segmentation tasks and dominate leader-boards of biomedical imaging processing challenges, including the segmentation of MS WMLs (Carass et al., 2017). Specifically, several convolutional neural network (CNN) architectures have been tailored for the segmentation of MS WMLs (Kaur et al., 2020). Some of them employ 2D convolutional layers (Aslani et al., 2019, Roy et al., 2018), whereas others employ 3D convolutional layers to incorporate information from all three spatial directions simultaneously (Hashemi et al., 2019, La Rosa et al., 2019, Valverde et al., 2017, Valverde et al., 2019). The clear edge these methods have over classical approaches is the capability of automatically extracting the relevant features for the task. Their application and generalization in clinical datasets, however, remains to be proved. They have often considered only 2D MRI sequences and segmentations were performed with a large minimum lesion volume threshold; for instance, Valverde et al. (2017) set this value to 20 voxels for the clinical MS datasets. Moreover, apart from (Valverde et al., 2019), all these deep learning methods are currently not publicly available. Finally, with the exception of our previous work (La Rosa et al., 2019), they have not been evaluated on CLs.

Compared to WMLs, imaging of CLs faces additional challenges due to their pathological features (Filippi et al., 2019). While WMLs can be automatically detected with high accuracy (Carass et al., 2017) from conventional MRI sequences, such as MPRAGE and FLAIR, CLs, which affect mostly the more superficial and less myelinated layers of the cortex (Filippi et al., 2019), have a low contrast to surrounding tissue with clinical MS MRI protocols. As mentioned above, the detection of CLs, at least at 3T, requires specialized imaging such as MP2RAGE and 3D DIR (Kober et al., 2012, Wattjes et al., 2007), and the number of lesions visible is still low in comparison to histopathology (about 20%) (Calabrese et al., 2010). These advanced imaging requirements limit the access to large training datasets. Furthermore, the automated detection of CLs is challenging as the number, volume, and location of CLs varies substantially across subjects (see Fig. 2).

To the best of our knowledge, only three studies explored the simultaneous segmentation of CLs and WMLs at 3T (Fartaria et al., 2016, Fartaria et al., 2017, La Rosa et al., 2019). Our first approach was based on a k-nearest neighbors (k‐NN) classifier (Fartaria et al., 2016), which was later on improved by including a partial volume tissue segmentation framework (Fartaria et al., 2017) to better delineate the lesions. In Fartaria et al. (2016)), we considered a multi-modal MRI framework including 3D MP2RAGE, 3D FLAIR, and 3D DIR sequences. Compared to manual segmentation, an overall WML and CL detection rate of respectively 77% and 62% was achieved. Recently, we have also explored the ability of deep learning architectures (La Rosa et al., 2019). We proposed an original 3D patch-wise CNN that improved lesion-wise results with respect to Fartaria et al., 2016). The main limitation of both approaches, however, was that training and testing them without the DIR sequence caused a significant drop in performance (CL detection rate from 75% to 58% in (La Rosa et al., 2019). Unfortunately, the DIR sequence is not widely acquired in clinics due to its long acquisition time (about 13 min) and frequent artefacts (Filippi et al., 2019), thus being for now mostly used for research purposes.

**Fig. 1:**
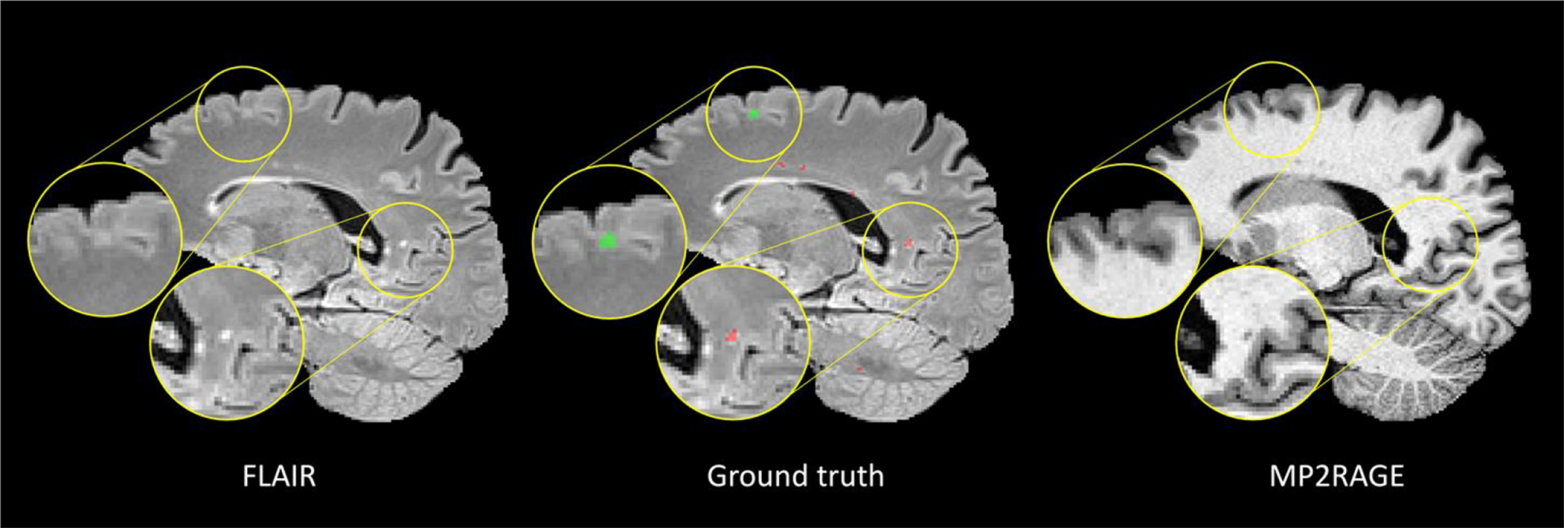
From left to right a sagittal slice of the FLAIR, manual lesion segmentation mask overlaid on the FLAIR, and MP2RAGE contrast. Colorcode of overlay: WMLs in red and CLs in green. The zoomed-in WML is clearly visible in FLAIR, whereas the MP2RAGE shows a higher contrast for the CL. (For interpretation of the references to colour in this figure legend, the reader is referred to the web version of this article.)

This study complements the literature with an evaluation of a deep learning method to segment CLs and WMLs based on two MRI sequences only (3D FLAIR and MP2RAGE, see Fig. 1) acquired at 3T. This choice reflects a clinically plausible set of input data that is not disruptive of established processes. Our aim is to provide a segmentation framework for different types of MS lesions, large and small, with a minimum lesion size of 3 voxels as recommended in the guidelines for MS CLs (Geurts et al., 2005). We propose a fully-convolutional architecture inspired by the 3D U-Net (Çiçek et al., 2016). Compared to our previous studies, we significantly extend our cohort of patients to 90 subjects from two different clinical centers. We evaluate the method firstly with a 6-folds stratified cross validation over the entire cohort and secondly with a train-test split.

## 2 Materials and methods

### 2.1 Datasets

**Fig. 2:**
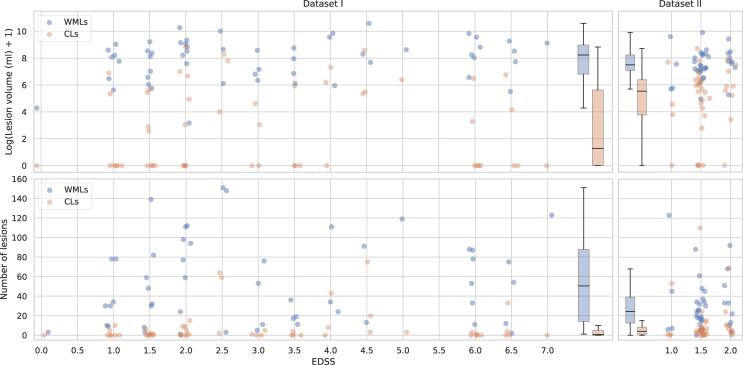
Distribution of the lesion volume and count per patient in our two datasets, considering WMLs and CLs separately. In the first row each patient has a blue dot corresponding to its WML volume and an orange dot for the CL one. In the second row this is repeated with the lesion count. Boxplots are added to summarize the distribution of each dataset. For visualization purposes jitter is added to the true EDSS value. (For interpretation of the references to colour in this figure legend, the reader is referred to the web version of this article.)

In this study, we consider two datasets for a total of 90 subjects overall. Dataset I includes patients at different stages of the disease, whereas Dataset II only at the early stages. Refer to Fig. 2 for an analysis of the differences between the datasets in terms of lesion volume and lesion count per patient.

#### 2.1.1 Dataset I

Images for Dataset I were acquired at Basel University Hospital from 54 patients (35 female / 19 male, mean age 44 ± 14 years, age range [22–73] years). According to the revised McDonald criteria (Thompson et al., 2018), 39 of these were diagnosed with relapsing remitting MS, 8 with primary progressive MS, and 7 with secondary progressive MS. Expanded Disability Status Scale (EDSS) scores ranged from 1 to 7 (mean 3.2 ± 1.9). Imaging was performed on a 3T MRI scanner (MAGNETOM Prisma, Siemens Healthcare, Erlangen, Germany) with a 64-channel head and neck coil. The following 3D sequences were acquired with a 1 mm^3^ isotropic spatial resolution: 3D-FLAIR (TR,TE,TI = 5000, 386, 1800 ms, acquisition time = 6 min), and a prototype MP2RAGE (TR,TE,TI1,TI2 = 5000, 2.98, 700, 2500 ms, acquisition time = 8 min). The study was approved by the Ethics Committee of our institution, and all patients gave written informed consent prior to participation.

#### 2.1.2 Dataset II

Images for Dataset II were acquired at Lausanne University Hospital from 36 patients (20 female / 16 male, mean age 34 ± 10 years, age range [20–60] years) diagnosed with relapsing remitting MS. Expanded Disability Status Scale (EDSS) scores ranged from 1 to 2 (mean 1.5 ± 0.3). Imaging was performed on a 3T MRI scanner (MAGNETOM Trio, Siemens Healthcare, Erlangen, Germany) with a 32-channel head coil. The following 3D sequences were acquired with a resolution of 1 × 1 × 1.2 mm^3^: 3D-FLAIR (TR,TE,TI = 5000, 394, 1800 ms, acquisition time = 6 min), and a prototype MP2RAGE (TR,TE,TI1,TI2 = 5000, 2.89, 700, 2500 ms, acquisition time = 8 min). The study was approved by the Ethics Committee of our institution, and all patients gave written informed consent prior to participation.

#### 2.1.3 Manual segmentation

WMLs appear as hyperintense areas in T2w images and as hypointense in T1w images and are usually well visible in conventional sequences at 3T. On the contrary, the majority of CLs cannot be clearly seen in FLAIR at 3T and specialized sequences as the MP2RAGE improve their detection (see Fig. 1, Fig. 6). In Dataset I, all lesions were detected and classified by consensus by a neurologist and a medical doctor with 11 and 5 years of experience in MS research, respectively. The medical doctor then manually segmented all lesions. In Dataset II both WMLs and CLs were manually detected and classified by consensus by the same neurologist who annotated Dataset I and one radiologist with 7 years of experience, using both imaging modalities. Their agreement rate prior to consensus was of 97.3%. The lesion borders were then delineated in each image looking at multiple planes by a trained technician. In total in our two datasets, 3856 WMLs and 728 CLs were manually labeled.

Furthermore, CLs were classified by a single expert in different subtypes according to (Calabrese et al., 2010). Cortical MS lesions can extend across the WM and GM (leukocortical, type I), can be contained entirely in the gray matter without extending to the surface of the brain or to the subcortical WM (pure intracortical, type II), or can be widespread from the outer to the inner layers of the cortex without perivenous distribution and often over multiple gyri (subpial, type III). Within our cohort, the majority (89%) of the cortical lesions identified belonged to type I, 11% to type II, and only 0.01% to type III. Given the high imbalance between different subtypes, in the automated segmentation analysis we pool all CLs together.

Lesions smaller than 3 voxels were automatically re-classified as background in the ground truth and in the predictions. This is equal to a volume of 3 µL for the lesions in Dataset I and 3.6 µL for the lesions in Dataset II. It should be noted that 3 µL corresponds to the consensus recommended minimum CL size in the 3D DIR sequence (Geurts et al., 2005), and we chose this as the minimum lesion size in our study as currently there is no guideline for the MP2RAGE. The distribution of the subject-wise total lesion volume and lesion count in our cohort can be seen in Fig. 2.

### 2.2 Methodology

**U-Net** Our network architecture is based on the 3D U-Net (Çiçek et al., 2016, Ronneberger et al., 2015). The U-Net architecture integrates an *analysis* path, where the number of feature maps are increased while the image resolution is being reduced, and a *synthesis* path where the resolution is increased and number of features decrease, yielding a semantic segmentation output. Several variants of it have been proposed, for example changing the resolution levels, varying the number of convolution layers or introducing residual blocks. The U-Net has been tested on different biomedical imaging segmentation applications, and methods based on it or on its 3D implementation (Çiçek et al., 2016) have won several segmentation challenges (Myronenko, 2018, Isensee and Maier-Hein, 2019). Two variants of the U-Net were also specifically proposed for MS WML segmentation (Kumar et al., 2019, Feng et al., 2018). Kumar et al. (2019) have proposed a dense 2D U-Net and showed promising results on a challenge dataset (Carass et al., 2017), even though the method was not compared to other deep learning approaches. Feng et al. (2018) have presented a standard 3D U-Net with advanced data augmentation, but again this work lacked an evaluation on a clinical dataset or a proper comparison with other state-of-the-art methods.

#### 2.2.1 Proposed architecture

**Fig. 3:**
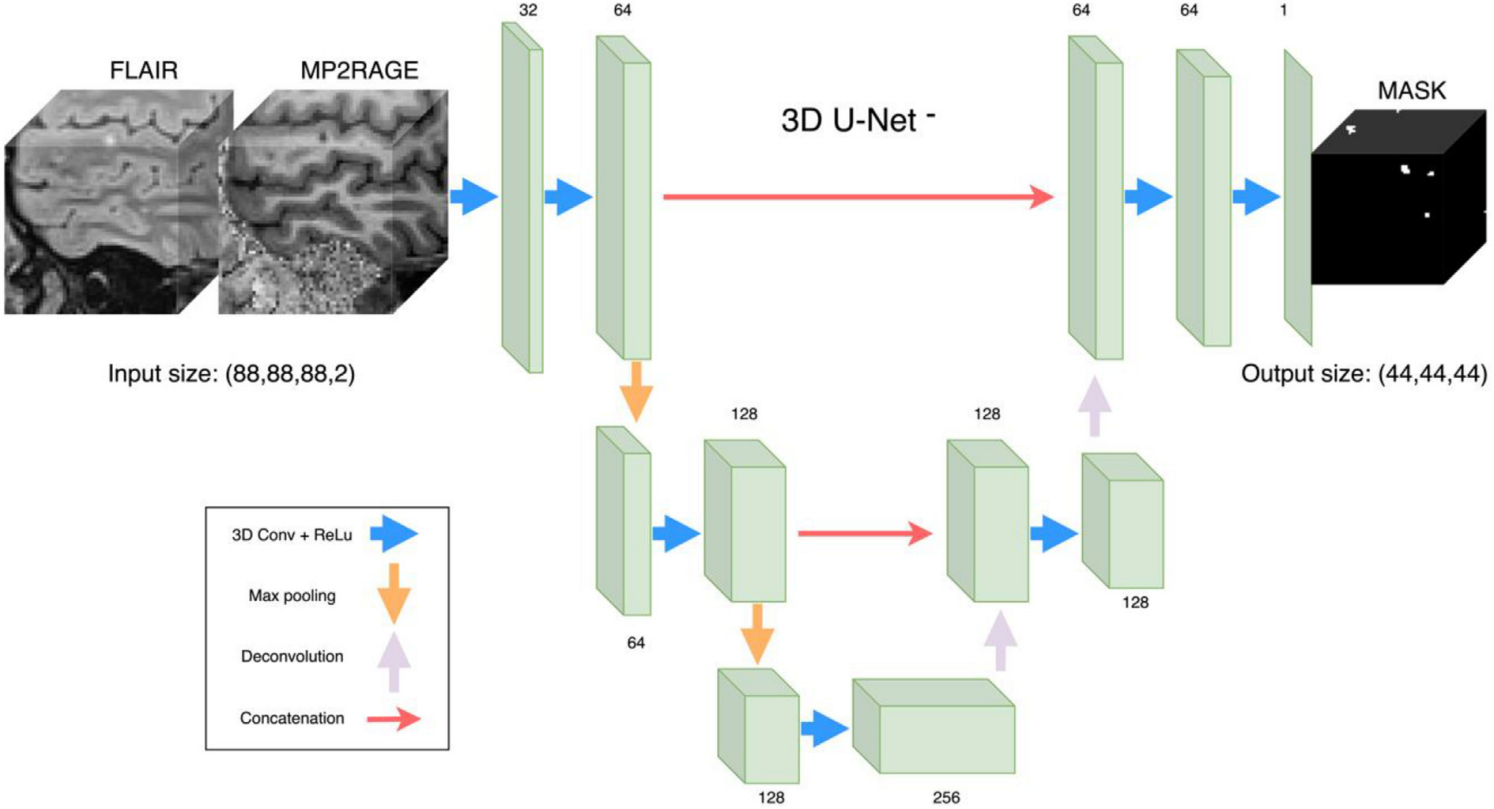
3D U-Net^-^ architecture proposed. On the left, examples of input patches in the two contrasts used, and on the right the relative lesion mask obtained in output.

Compared to the original implementation of the U-Net, we reduce the number of resolution levels from four to three (therefore 3D U-Net**^-^**) because of the limited number of available CLs. By doing so, we drastically reduce the number of trainable parameters from 18.3 M to 3.8 M and thus decrease the risk of overfitting. Furthermore, our choice of a 3D architecture is motivated by the fact that the input modalities FLAIR and MP2RAGE are both 3D acquisitions, and therefore we want to fully exploit the volumetric anatomical information. The 3D U-Net**^-^** architecture integrates a spatial context of 41 voxels. In our experiments, we use an input shape of (88, 88, 88) and because no padding is applied through the network the output size was (48, 48, 48) (see Fig. 3). In the analysis path, the 3D convolutional filters (each followed by a ReLu activation function) has respectively 32, 64, 64, 128, and 128 filters. The decoder has the following number of feature maps: 256, 128, 128, 64, 64, 1. Skipped connections are present as in the original implementation (Çiçek et al., 2016).

#### 2.2.2 Pre-processing

The only pre-processing step performed is a rigid registration based on mutual information of the FLAIR image of each subject to the corresponding MP2RAGE (UNI contrast) image with ELASTIX (Klein et al., 2010). Each multi-step pre-processing pipeline, for instance, skull stripping and/or bias field correction has its own data dependent effect on the performance. While we cannot exclude the possibility that a certain combination of pre-processing steps would lead to better performance for a given data set, we reduce the model-related sources of variance in performance to the network architecture and its training, which leads to an integrated solution that facilitates evaluation and incremental improvements.

#### 2.2.3 Training

Prior to training all input volumes are normalized with zero mean and unit variance. We implement a sampling strategy by which each connected component in the ground truth has the same possibility of being sampled, regardless of its size. Thus, we encourage the network to focus also on the smaller structures such as CLs. L2 regularization was used with a regularization factor of 1e-5. The network is trained with a pixel-wise weighted cross-entropy loss function with the following weights: background 1, WMLs 1, CLs 5. This is motivated by the fact that there are about 5 times less CLs than WMLs in each fold of our dataset. The learning rate is initially set to 1e-8 and gradually increased in the first 2000 iterations to 1e-4 in a warm-up phase. Afterwards it is reduced by half every 10,000 iterations. The batch size is set to 2, and Adam is used as optimizer. The validation loss is monitored with early stopping to determine when to stop the training.

#### 2.2.4 Data augmentation

Data augmentation is a well-known technique for deep neural networks to increase the performance in the testing set. In our work, extensive data augmentation is performed on-the-fly to prevent overfitting. The transformations are carefully chosen as, since we are dealing with large 3D patches, excessive augmentation would slow down the training. More specifically, we apply random rotation of the input volumes around the z axis only up to 90°, random spatial scaling up to 5% of the volume size, and random flipping along all three axes.

#### 2.2.5 Implementation

The code is implemented in the Python language in the NiftyNet framework (Gibson et al., 2018) based on TensorFlow (Abadi, 2016). The software requires Python version 3.6. Training also requires CUDA/cuDNN libraries by NVIDIA and compatible hardware. The code is publicy available along with a trained model^1^.

### 2.3 Evaluation

#### 2.3.1 Comparison with other related methods

For comparison, we evaluated two state-of-the-art MS WML segmentation methods publicly available:

• LST-LGA is an unsupervised lesion growth algorithm (Schmidt et al., 2012) implemented in the LST toolbox version 3.0.0 (LST, 2020) for Statistical Parametric Mapping (SPM). LST-LGA has been widely evaluated in the context of MS WML segmentation and used as comparison with more recent approaches (Aslani et al., 2019, Valverde et al., 2017, Roy et al., 2018). In a nutshell, the algorithm performs an initial bias field correction and affine registration of the T1 image (in our case the MP2RAGE) to the FLAIR, and then proceed with the lesion segmentation. Lesions are identified based on a voxel-wise binary regression with spatially varying parameters. We applied LST-LGA with the default initialization parameters (kappa = 0.3, MRF = 1, maximum iterations = 50). The final threshold to obtain a binary segmentation mask was optimized in the validation set by maximizing the dice coefficient.
• nicMSlesions is a state-of-the-art deep learning WML segmentation method (Valverde et al., 2017, Valverde et al., 2019). Having reached an excellent performance in a MS lesion segmentation challenge (Carass et al., 2017), it is now a common method to compare with (Aslani et al., 2019, Weeda et al., 2019, Roy et al., 2018). This method selects lesion candidates’ voxels based on the FLAIR contrast and extracts 11x11x11 patches around them. A double CNN is then trained to first find lesion candidates and then reduce the false positive rate. The pipeline includes as pre-processing steps a registration of the different modalities to the same space, skull-stripping, and denoising. This approach was run with all the default parameters, including a maximum of 400 epochs, early stopping, and the patient value set to 50 epochs.

#### 2.3.2 Evaluation strategies and metrics

**Fig. 4:**
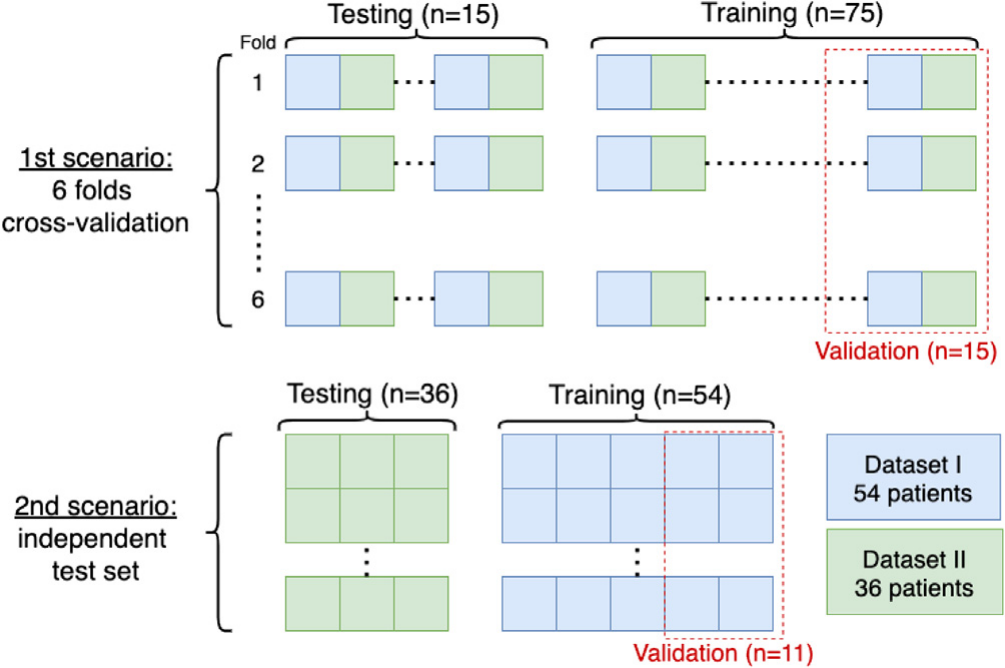
Scheme of the two evaluation scenarios.

We evaluated the three methods in two different scenarios (see Fig. 4). First, we performed a stratified 6-folds cross validation pooling data from Dataset I and II. Second, we trained the supervised methods on the subjects of Dataset I and evaluated using data from Dataset II.

Results were evaluated quantitatively with the manually delineated masks. We computed the following, widely used (Carass et al., 2017, Kaur et al., 2020, Lladó et al., 2012), evaluation metrics: dice coefficient (DSC), absolute volume difference (AVD), voxel-wise positive predicted value (PPV), lesion-wise true positive rate (LTPR), lesion-wise false positive rate (LFPR), WML detection rate (LTPR_WM), CL detection rate (LTPR_CL) as defined here (Carass et al., 2017).

Statistical analysis was also performed at the patient-wise level using the SciPy Python library (SciPy 1.0 Contributors et al., 2020). As the distributions violate the normality assumptions, Wilcoxon signed rank test was used to statistically test differences in LTPR_WM, LTPR_CL, and LFPR. All tests were adjusted for multiple comparison using a Bonferroni correction. Statistical differences were considered for p-value < 0.05. We computed the Pearson's linear correlations between manual and estimated masks to analyze the volume differences.

## 3 Results

### 3.1 Cross-validation

**Fig. 5:**
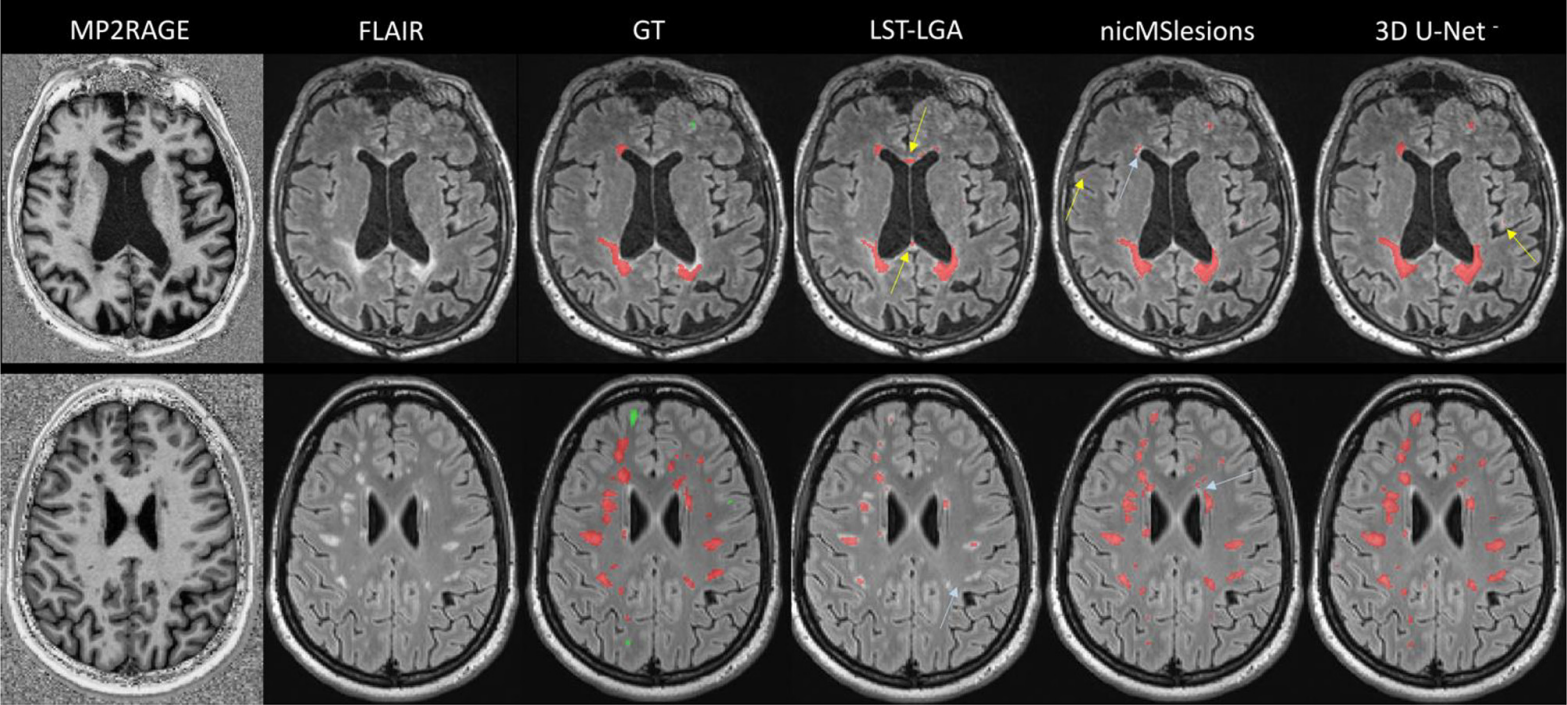
Visual illustration of results for all three methods. In the first row is presented a subject from Dataset I, and in the second row a subject from Dataset II. Colorcode of overlay of the ground truth (GT): WMLs in red and CLs in green. The yellow arrows point at false positives of the automatic methods, whereas the light blue arrows at false negatives. (For interpretation of the references to colour in this figure legend, the reader is referred to the web version of this article.)

**Fig. 6:**
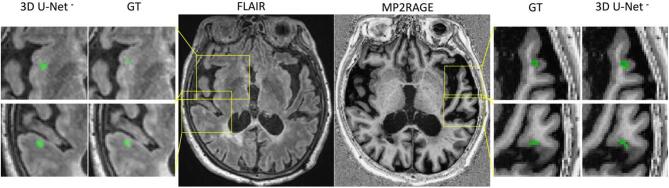
Four examples of correctly detected cortical lesions by our proposed method. All lesions are better visible in the MP2RAGE sequence compared to the FLAIR.

There were 75 training cases from which 20% (15 patients) were used as validation set to determine early stopping and optimize the threshold and 15 testing cases in each fold (see Fig. 4). The threshold was chosen as the value (with intervals of 0.05) that gave the highest dice coefficient in the validation set. Qualitative results are reported in Fig. 5, where the first row shows a slice of a subject from Dataset I, and the second row one from a subject of Dataset II. Moreover, in Fig. 6 we show examples of CLs correctly detected by our proposed 3D U-Net**^-^**.

The median of all metrics obtained in the test folds of the cross-validation are shown in Table 1. 3D U-Net**^-^** achieves a 75% CL detection rate and the best performance in all metrics except for the PPV, for which LST-LGA has the best value (71%). Overall, the results of 3D U-Net**^-^** and nicMSlesions are in line with other recent works, both for WMLs (Carass et al., 2017) and for CLs (Fartaria et al., 2016, La Rosa et al., 2019).

**Table 1:** Median values (IQR) of the metrics obtained for the different methods on the cross-validation evaluation (90 subjects). The minimum lesion size is 3 voxels. The last column shows the inference time. The best result per each metric is shown in bold.

Method	Dice	AVD	PPV	LTPR	LTPR_WM	LTPR_CL	LFPR	Time (s)
LST-LGA	0.36 (0.19)	0.60 (0.31)	**0.71** (0.34)	0.36 (0.24)	0.38 (0.25)	0.10 (0.33)	0.36 (0.35)	370
nicMSlesions	0.53 (0.28)	0.32 (0.43)	0.52 (0.43)	0.65 (0.26)	0.67 (0.26)	0.53 (0.39)	0.45 (0.40)	430
3D U-Net**^-^**	**0.62** (0.16)	**0.27** (0.30)	0.61 (0.23)	**0.76** (0.20)	**0.77** (0.22)	**0.75** (0.50)	**0.29** (0.25)	**20**

**Fig. 7:**
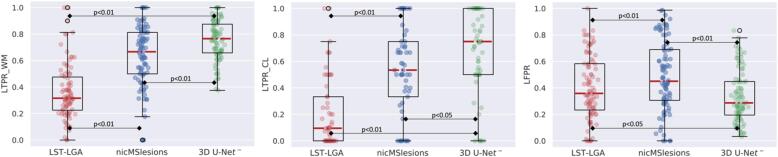
Boxplots of the patient-wise metrics obtained for the three methods. The p-values are computed with the Wilcoxon signed rank test.

We further analyzed the patient-wise LTPR and LFPR by the boxplots in Fig. 7. 3D U-Net**^-^** significantly outperforms the other two methods in the three detection accuracy metrics (refer to Fig. 7 for the p-values). Moreover, there is only a slight difference between the 3D U-Net**^-^** CL and WML detection rate.

**Fig. 8:**
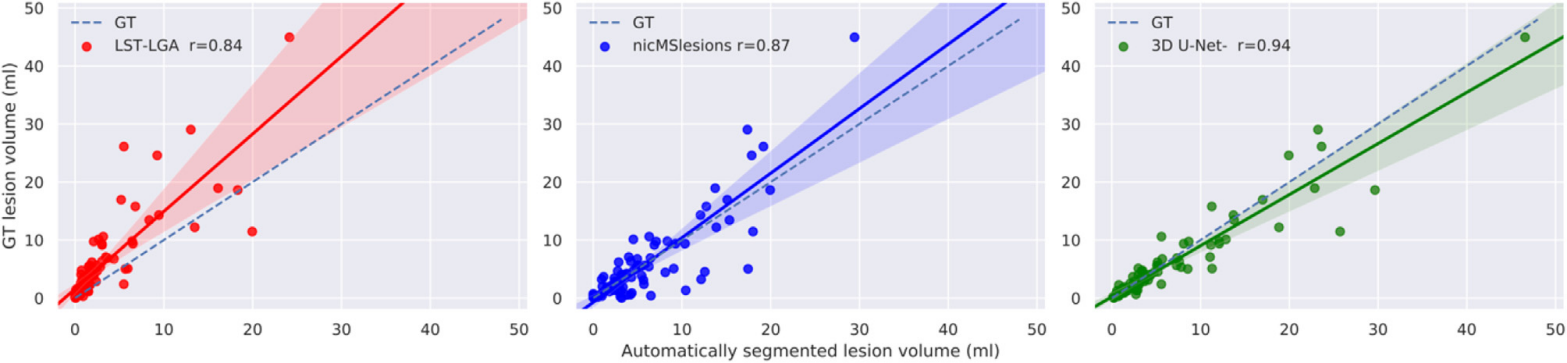
Correlation between the manual lesion volume and the automatically segmented one. The solid lines show the linear regression model between the two measures along with confidence interval at 95%. The dashed lines indicate the expected lesion volume estimates. The Pearson's linear correlation coefficient between manual and automatic lesion volume is reported in the legend (r = 0.84 for LST, r = 0.87 for nicMSlesions, r = 0.94 for 3D U-Net^-^.

Fig. 8 shows the correlation between the manually segmented lesion volumes and the ones automatically estimated. On the identity lines the predicted and estimated volumes are equal an the closer the results are to the line, the more accurate. The solid lines show the linear regression model fitted with these points, and the Pearson's linear correlation coefficient is reported for each method in the legend.

**Fig. 9:**
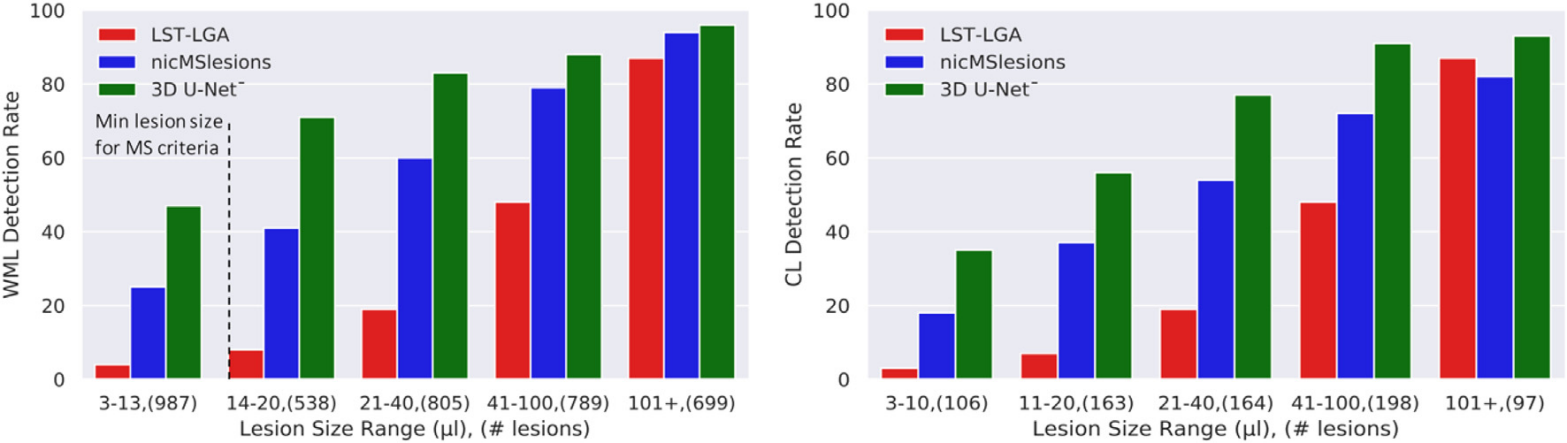
Detection rate for WMLs and CLs for the three methods evaluated considering different lesion size ranges. In parenthesis are reported the total number of lesions of each range. The dashed line shows the minimum lesion size established for WMLs.

We also analyzed the results per lesion size range. As mentioned above, we decided to stay below the MS WML minimum size recommended in the diagnostic criteria, as our datasets include also CLs. In Fig. 9 is presented the detection rate for WMLs and CLs separately for the three methods.

### 3.2 Independent test set

In the second scenario we aim at exploring the generalization of our segmentation framework by training on one single center while testing on the other one (see Fig. 4). We trained a model on Dataset I, keeping 20% of the cases for validation (n = 11), and evaluated the performance on the independent data of Dataset II. This setting simulates a more realistic scenario where our method is evaluated with imaging data acquired outside the training site. Moreover, let us note that patients in Dataset II are at very early stages of MS with EDSS scale ranging between 1 and 2 only (see Fig. 2).

**Fig. 10:**
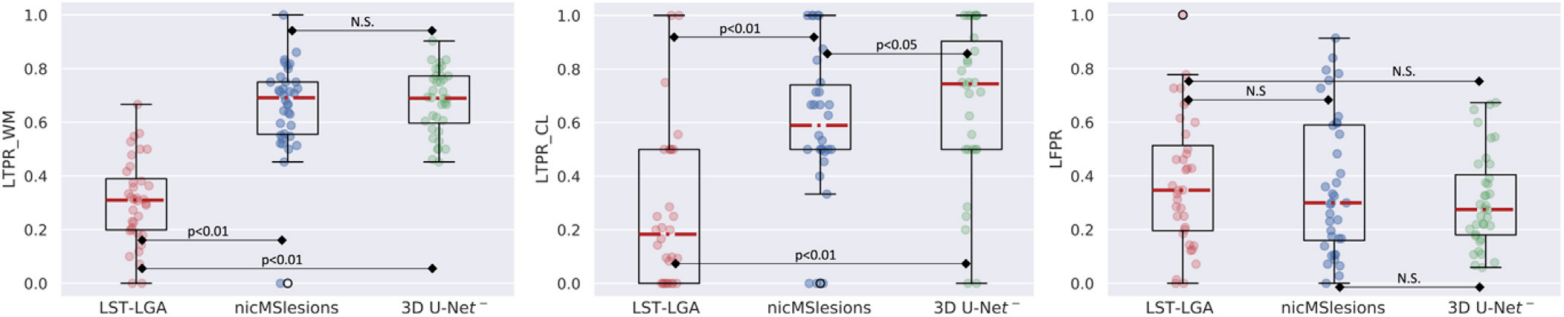
Boxplots of the patient-wise metrics obtained for the three methods in the separate test set. The p-values are computed with the Wilcoxon signed rank test. N.S.: not significant.

In Table 2 we report the quantitative criteria evaluated on an independent test set. Our proposed method achieves a 71% CL detection rate and outperformed the others in all metrics except for the PPV and the LTPR_WM, for which it achieves the best results (69%) together with nicMSlesions. Finally, in Fig. 10 we show the boxplots for the WM and CL LTPR, and the LFPR, with their relative significant differences performed with the Wilcoxon signed rank test.

**Table 2:** Median values (IQR) of the metrics obtained for the different methods on the independent test set (36 subjects). The minimum lesion size is 3 voxels. The last column shows the inference time. The best result per each metric is shown in bold.

Method	Dice	AVD	PPV	LTPR	LTPR_WM	LTPR_CL	LFPR	Time (s)
LST-LGA	0.32 (0.27)	0.63 (0.25)	**0.75** (0.29)	0.28 (0.21)	0.30 (0.19)	0.19 (0.20)	0.35 (0.34)	370
nicMSlesions	0.58 (0.18)	0.27 (0.21)	0.62 (0.26)	0.67 (0.16)	**0.69** (0.20)	0.59 (0.27)	0.31 (0.37)	430
3D U-Net**^-^**	**0.60** (0.19)	**0.13** (0.27)	0.64 (0.24)	**0.69** (0.10)	**0.69** (0.12)	**0.71** (0.48)	**0.27** (0.33)	**20**

## 4 Discussion

With a simple deep learning architecture that was trained end-to-end and did not include explicit feature engineering or advanced pre-processing, we segmented CLs and WMLs using one conventional and one specialized MRI contrast with a detection rate of 76% (median) and lesion false positive rate of 29% (median). The proposed prototype 3D U-Net**^-^** outperformed the baseline methods and proved to generalize well for cases acquired with a different scanner. Consequently, we foresee our proposed method as a useful support tool in a research setting where MS CLs and WMLs need to be segmented in a high number of MRI scans. The automatic segmentation obtained could, for instance, represent a first lesion labelling to then be refined by the experts, allowing them to further standardize and speed up the overall process.

As reflected by being recently introduced in the MS diagnostic criteria (Thompson et al., 2018), CLs are of great clinical interest, yet their automated segmentation has been receiving little attention. The fully-convolutional 3D U-Net**^-^** has a relatively low number of training parameters (3.8 M) and it is fast to run at inference time (about 20 s to infer a new subject, not counting initial intra-subject registration). The method was evaluated on two datasets of 54 and 36 subjects, respectively. In order to emulate a realistic clinical setting, we considered a minimum lesion size of 3 voxels, which is the recommended minimum size of CLs for 3D sequences with at least 1 *mm* voxel spacing (Geurts et al., 2005). This is smaller than what previous automatic studies have reported for WM lesions (20 voxels in (Valverde et al., 2017), for instance), and also much smaller than the clinical definition of minimum WML diameter of 3 *mm* (Grahl et al., 2019, Thompson et al., 2018) corresponding in a spherical approximation to a volume of about 14 *mm^3^*.

We compared our proposed approach with two reference methods: an unsupervised lesion growth approach (LST-LGA) and a supervised deep learning technique (nicMSlesions), both of which were originally proposed for MS WML segmentation only. Our first evaluation consisted in a 6-folds per site stratified cross validation including all 90 subjects. In this way, we evaluated the performance over a large dataset, with significant variability of lesion count and volume across subjects. We considered the main metrics evaluated in MS lesion segmentation challenges (Carass et al., 2017). In particular, we focused our study on the CLs and WMLs detection rate, as well as the absolute volume difference, because lesion count and volume are included in the MS diagnostic criteria (Thompson et al., 2018). Among the metrics reported, the PPV is a pixel-wise metric that by definition ignores the false negatives, which in our case represent the missed lesions, and are therefore quite important. Moreover, it should be noted that the widely used dice coefficient is not reflecting well the overall performance in the case of very small structures as in our study. For how it is computed, the dice is naturally biased towards the lesion, penalizing cases with only small structures more severely.

Table 1 shows that 3D U-Net**^-^** outperformed the other methods in all metrics except for the PPV. The high detection rate for both WMLs and CLs proves the capability of our proposed method to detect both types of MS lesions with similar accuracy, even if the latter have a low contrast in the FLAIR images (see Fig. 1, Fig. 5). Performing Wilcoxon signed rank test using a Bonferroni correction for multiple comparisons, 3D U-Net**^-^** is significantly better than the other approaches in CL and WML detection rate and in false positive rate. Our claims are also supported by an analysis of the correlation between the manual total lesion volume and the one automatically segmented (see Fig. 7). Moreover, we explored the lesion detection rate per lesion size (Fig. 8). By increasing the minimum lesion size all methods perform better, and their relative difference in detection rate decreases.

In the independent test scenario, we trained the two supervised methods with the patients of Dataset I and tested the models with the subjects of Dataset II. In this way, we evaluated the methods on cases acquired in another site and with a different scanner. We acknowledge, however, that the acquisition parameters of both scanners were very similar, thus limiting the generalization to this particular setting. It can be observed in Table 2 that also in this independent test setting, the supervised deep learning approaches outperform LST-LGA in terms of lesion detection, dice coefficient, and also volume difference. NicMSlesions significantly improves with respect to the previous scenario its false positive rate (p-value < 0.01) and reaches the best WM LTPR together with 3D U-Net**^-^** (69%). Thus, these results support previous claims of generalizing very well in WML segmentation to cases from different datasets (Valverde et al., 2019, Weeda et al., 2019). Moreover, let us note that in this study we assessed nicMSlesions considering a much smaller minimum lesion size than in any other previous study where it was tested (Valverde et al., 2017, Valverde et al., 2019, Weeda et al., 2019). Compared to a 69% WML detection rate, nicMSlesions CL detection rate was only 59%. We hypothesize this is due to the dependency of its lesion candidate selection on the FLAIR intensity value. As the sensibility of FLAIR to CLs is limited, several CLs might be discarded for this reason. Interestingly, 3D U-Net**^-^** performs even better than in the first scenario in terms of volume difference (p-value < 0.05), but understandably performs slightly worse in cortical and white matter LTPR (p-value N.S.). In particular, it reaches the same result as nicMSlesions (69%) in terms of WMLs, but it performs significantly better for the CLs detection rate (71% vs 59%, p-value < 0.05, Wilcoxon signed rank test). Furthermore, is worth noting that in this scenario the ground truth of the training and testing datasets was delineated by different experts. This might contribute to intrinsic differences in the labeled masks, thus posing additional challenges to the automatic methods.

A major advantage of our CL and WML segmentation framework is that it is based on two 3T MRI sequences only. As successfully done in previous automatic segmentation studies (Fartaria et al., 2016, Fartaria et al., 2017, La Rosa et al., 2019), we explore the use of the specialized MP2RAGE sequence instead of the conventional MPRAGE. Nonetheless, we acknowledge that this sequence is still not widely acquired in clinical routine for MS. However, in order to visually detect CLs, specialized sequences are needed (Calabrese et al., 2010, Filippi et al., 2019), and the MP2RAGE is a promising substitute of the conventional MPRAGE. MP2RAGE has increased lesion and tissue contrast compared to the common MPRAGE (Kober et al., 2012). Moreover, while currently the MP2RAGE sequence requires about 8 min of scanning time protocol, recent developments have shown that its acquisition time can be reduced to less than 4 min without compromising the image quality (Mussard et al., 2020). Thus, it could easily be included (additionally to or instead of the MPRAGE) in a 3T MRI MS clinical protocol in order to support the CLs analysis.

In contrast to other studies, we report the detection accuracy of very small lesions that when evaluated using an overlap measure, such as Dice coefficient, and in presence of large lesions would not contribute strongly to the performance evaluation but may be clinically relevant. However, our method detected very small lesions (between 3 and 10 voxels, 3–10 µL) poorly. We believe this is due to partial volume effect and artefacts affecting them and could be improved if more small lesions would be included in the training dataset. In our study, experts agreed by consensus on the lesion detection, but the ground truth masks of each dataset were manually delineated only once and by different experts. This limits our analysis, not allowing us to compare the automatic methods' performance to the inter and intra rater reliability. Moreover, given the 3T MRI settings of our work, the vast majority of CLs detected by the experts (89%) are leukocortical/juxtacortical. Thus, also the accurate CL detection of our proposed method is limited to this particular CL type.

In conclusion, we achieve an accurate CLs and WMLs segmentation with a simple 3D fully-convolutional CNN, which operates on data that is not treated with advanced pre-processing, is fast to run at inference time and generalizes well across two different scanners. The considered MRI sequences are close to the ones of a clinical scenario, meaning that the proposed approach could support experts in the lesion segmentation process. Future work will aim at improving the lesion delineation. This might include, for instance, exploring the T1 map acquired together with the MP2RAGE sequence. Moreover, we will tackle the challenging task of providing an output segmentation that classifies the lesions in WMLs and CL types, as this could have an added clinical value.

## CRediT authorship contribution statement

**Francesco La Rosa:** Conceptualization, Methodology, Software, Validation, Formal analysis, Writing - original draft, Writing - review & editing, Visualization. **Ahmed Abdulkadir:** Conceptualization, Methodology, Software, Data curation, Writing - original draft, Writing - review & editing. **Mário João Fartaria:** Conceptualization, Resources, Data curation, Writing - original draft, Writing - review & editing. **Reza Rahmanzadeh:** Resources, Data curation, Writing - original draft. **Po-Jui Lu:** Resources, Data curation, Writing - review & editing. **Riccardo Galbusera:** Resources, Data curation, Writing - original draft. **Muhamed Barakovic:** Resources, Data curation, Writing - original draft. **Jean-Philippe Thiran:** Writing - original draft, Supervision, Project administration, Funding acquisition. **Cristina Granziera:** Resources, Data curation, Writing - original draft, Writing - review & editing, Project administration. **Merixtell Bach Cuadra:** Conceptualization, Writing - original draft, Writing - review & editing, Supervision, Project administration, Funding acquisition.

## Declaration of Competing Interest

Mário João Fartaria is employed by Siemens Healthcare AG. The other authors have nothing to declare.

## Footnotes

1 La Rosa, Francesco, Abdulkadir. Ahmed, Thiran, Jean-Philippe, Granziera, Cristina, & Bach Cuadra, Merixtell. (2020, July 7). Software: Multiple sclerosis cortical and WM lesion segmentation at 3T MRI: A deep learning method based on FLAIR and MP2RAGE (Version v1.0). Neuroimage: Clinical. Zenodo. http://doi.org/10.5281/zenodo.3932835
